# Effect of Mineral Fertilization on Vegetation of HNV Pastures in the Apuseni Mountains (Romania)

**DOI:** 10.3390/plants14233564

**Published:** 2025-11-21

**Authors:** Ioana Ghețe, Ioan Rotar, Anca Pleșa, Alexandru Ghețe, Claudiu Șerban, Vlad Stoian

**Affiliations:** 1 Department of Grasslands and Forage Crops, Faculty of Agriculture, University of Agricultural Sciences and Veterinary Medicine Cluj-Napoca, Calea Mănăstur 3-5, 400372 Cluj-Napoca, Romania; ioan.rotar@usamvcluj.ro (I.R.); anca.plesa@usamvcluj.ro (A.P.); 2 Department of Technical and Soil Sciences, Faculty of Agriculture, University of Agricultural Sciences and Veterinary Medicine Cluj-Napoca, Calea Mănăstur 3-5, 400372 Cluj-Napoca, Romania; alexandru.ghete@usamvcluj.ro; 3 Research and Development Institute for Montanology, 557085 Cristian, Romania; 4 Department of Microbiology, Faculty of Agriculture, University of Agricultural Sciences and Veterinary Medicine Cluj-Napoca, Calea Mănăştur 3-5, 400372 Cluj-Napoca, Romania; vlad.stoian@usamvcluj.ro

**Keywords:** mountain grasslands, long-term experiment, fertilizer gradient, diversity, *Festuca rubra*, *Agrostis capillaris*, sustainable management

## Abstract

High-Nature-Value (HNV) grasslands in the Apuseni Mountains represent traditional semi-natural ecosystems with high biodiversity and major ecological value, but are constantly exposed to pressures generated by both agricultural intensification and abandonment. This study asses the effects of long-term mineral fertilization on floristic composition and grassland diversity within a permanent experiment initiated in 2001 in Gârda de Sus (Romania). Four variants of mineral fertilization were tested: control (N0), low-input (N_50_P_25_K_25_), medium-input (N_100_P_50_K_50_) and high-input (N_150_P_75_K_75_). Floristic analyses were performed using the modified Braun-Blanquet method, and the data were interpreted using multivariate analyses and diversity indices. The results revealed a clear trophic gradient. Both the control and low-input variants maintained high diversity and the stability of communities dominated by *Festuca rubra* and its associated oligotrophic species. In contrast, medium and high fertilization produced a pronounced reduction in community components, with the dominance of nitrophilous species, especially *Agrostis capillaris*, a species which is consumed by animals. Multivariate analyses confirmed that the dominant effect on plant communities was the fertilization level, outweighing the interannual climatic variations. Low fertilization maintained biodiversity with minimal changes after 17 years, while higher inputs led to significant species losses and reduced stability of community. The results provide a scientific benchmark for creating specific sustainable management scenarios and highlight the need for accepted organic alternatives. This study is one of the few long-term experiments in the Carpathians that documents the impact of fertilization on HNV grasslands and provides essential benchmarks for adapting management strategies in the context of the Common Agricultural Policy.

## 1. Introduction

High-Nature-Value (HNV) grasslands are an essential element of the European rural landscape, recognized for their high biodiversity, their role in maintaining ecological balance and the cultural and economic value they generate [1,2,3]. In the European Union, HNV grasslands occupy significant areas, especially in mountain and hilly areas, where traditional agricultural practices have favored the conservation of floristic diversity [4,5,6,7]. At the same time, the application of traditional management on the grasslands has created specific landscape elements that give the cultural landscape its own identity [8,9]. In Romania, they are especially widespread in the Carpathians, being considered cores of agriculture with high natural value and being integrated into the Natura 2000 network [10,11].

These ecosystems provide a wide range of services. As provisioning services, they provide high-quality forage for ruminants that is rich in proteins and minerals [12,13,14]; medicinal plants of pharmaceutical interest, such as *Arnica montana*, emblematic of the Apuseni [15,16]; and resources for beekeeping. At the level of regulatory services, these ecosystems contribute to carbon sequestration, water regime regulation and climate change mitigation [17,18,19]. In the category of supporting services, HNV grasslands maintain soil fertility and nutrient cycling, supporting the resilience of agroecosystems [20,21]. At the same time, this type of HNV ecosystem has a cultural and social role, being appreciated for its landscape value, agro-pastoral traditions and tourism potential [22,23].

However, HNV grassland ecosystems are exposed to multiple and major threats. Agricultural intensification, through excessive fertilization and mechanization, leads to the simplification of the vegetation cover and the reduction in biodiversity [24,25,26]. In contrast, agricultural abandonment favors secondary succession processes, with the establishment of woody species and the reduction in forage value [27,28,29,30]. This process reduces species richness and causes significant changes in the structure of plant communities, including the loss of herbaceous species with high forage value in favor of less valuable ones [31]. In many European regions, the abandonment without any management of interventions favors the invasion of woody species, leading to the loss of open habitats and the degradation of biodiversity [32]. Climate change acts as a multiplier factor, modifying the hydrological and thermal regime and accelerating the transformation of these ecosystems [33,34]. In addition, both socio-economic and land use changes influence the resilience of cultural landscapes, a phenomenon that is visible in the entire area of Carpathians [35,36,37].

In this context, mineral fertilization has a dual and opposite role [38,39]. On the one hand, it supports increased productivity and improves the nutritional value of the feed [40,41,42]. On the other hand, high doses and repeated applications reduce floristic diversity, favor nitrophilous species and compromise ecosystem stability [43]. The studies on the application of mineral fertilizers confirm that the balance between productivity and biodiversity depends on the dosage, frequency of application and the ecological context [44,45]. Additionally, the effects of fertilization are not only manifested on vegetation, but also on the soil microbiome, where significant functional changes occur in the symbiotic status of species [46,47,48].

Long-term experiments provide essential benchmarks for understanding the critical thresholds at which meadow and pasture ecosystems change their structure and functionality [49,50,51]. Especially for those located in mountain areas, where socio-economic and climatic pressures are increasing, such research is indispensable for the substantiation of new and sustainable management strategies [52,53,54]. The integration of modern vegetation analysis methods [55,56,57] and research on alternative management uses, such as mulching [58,59,60], contributes to the development of a complex and interdisciplinary assessment framework and supports the decision-making process regarding the sustainable management of mountain resources.

The present study aims to evaluate the effects of long-term mineral fertilization on HNV meadows in the Apuseni Mountains, analyzing changes in floristic composition, diversity and types of plant communities 17 years since its establishment. Long-term experiments have a high scientific value, as they allow us to explore series of consistent and diverse data, which reflect the evolutions of meadow ecosystems, which are increasingly vulnerable. The main objectives of the study are proposed to analyze in detail species and community changes due to long-term application of fertilizers. Specifically, we aimed to evaluate the changes that occurred in the composition of plant communities, identify and interpret the trends of change associated with each treatment applied as well as to assess the specific responses of species to these treatments.

## 2. Results

### 2.1. Cluster Analysis of Vegetation Under the Influence of Mineral Fertlization

To characterize the floristic composition of the meadow during the study period, the surveys carried out in the three experimental years (2015–2017) were analyzed. In the floristic composition of the experimental variants, 43 plant species were identified, belonging to several botanical families characteristic of meadow ecosystems. The synthesized floristic data are presented in the form of average abundance–dominance (ADM) values, calculated for each repetition, for the three years, considering that the complete data set includes 48 individual surveys (16 for 2015, 16 for 2016 and 16 for 2017), resulting in the analysis of a very large volume of information which would make consultation and interpretation difficult. The presentation of mean values (ADM) represents a scientifically relevant approach, providing a clear and comparable picture of the vegetation of the floristic composition in the three experimental years. The table with ADM values is available in the Supplementary Files (Supplementary Table S1). Cluster analysis, based on the Sørensen index (Bray–Curtis) and the UPGMA method, highlighted the separation of the floristic observations into four major groups that correspond to experimental treatments with different levels of mineral fertilization. Cluster 1 (V1—control): dominated by *Festuca rubra—Agrostis capillaris*, with high diversity and the presence of oligotrophic species characteristic of high-natural-value mountain grassland. Cluster 2 (V2—N_50_P_25_K_25_): characterized by the community of *Trisetum flavescens—Agrostis capillaris*, indicating a trend of change in community structure towards species with moderate nutrient requirements. Cluster 3 (V3—N_100_P_50_K_50_): dominated by *Agrostis capillaris—Trisetum flavescens* as a secondary species, which reflects the fertilization pressure on species with high forage value. Cluster 4 (V4—N_150_P_75_K_75_): accentuated simplification of phytocenosis, with the almost exclusive dominance of *Agrostis capillaris*, a nitrophilic species tolerant to high doses of nitrogen.

**Figure 1:**
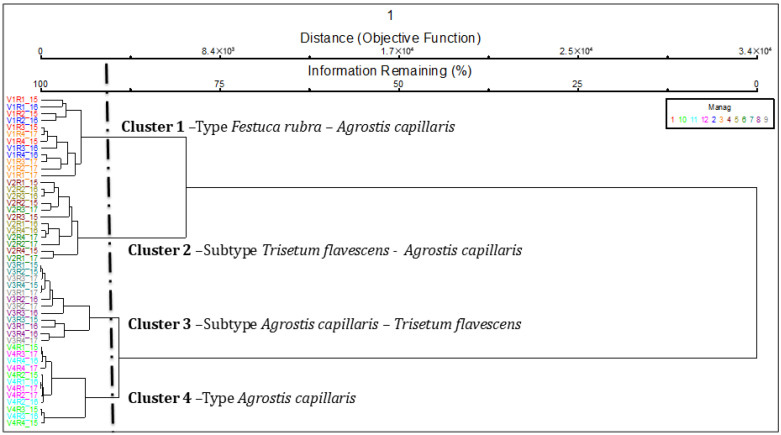
Dendrogram of plant community types. Legend: Manag—management; V1—control; V2—N50P25K25; V3—N_100_P_50_K_50_; V4—N_150_P_75_K_75_; 15, 16, 17 = the three experimental years (2015, 2016, 2017); R1, R2, R3, R4 = the four replications. Management codes: 1, 2, 3—V1; 4, 5, 6—V2; 7, 8, 9—V3; 10, 11, 12—V4. First management correspond to year 2015, second management correspond to year 2016 and third management correspond to year 2017.

When cutting the dendrogram at ~90% remaining information (PC-ORD), two major groups are obtained (Figure 1). Group 1 (clusters 1–2) brings together two types of communities characterized by the *Festuca rubra—Agrostis capillaris* floristic assemblage, with the emergence of a transitional group towards moderate trophic requirements under low-input fertilization (V2: N_50_P_25_K_25_ described here as *Trisetum flavescens—Agrostis capillaris*). Group 2 (clusters 3–4) is dominated by *Agrostis capillaris* and corresponds to treatments with higher intensity: an *Agrostis capillaris—Trisetum flavescens* assemblage under medium input (V3: N_100_P_50_K_50_) and a simplified *Agrostis capillaris* community under high input (V4: N_150_P_75_K_75_).

### 2.2. Spatial Distance in Plant Community Projection Due to Long-Term Fertilization

**Figure 2:**
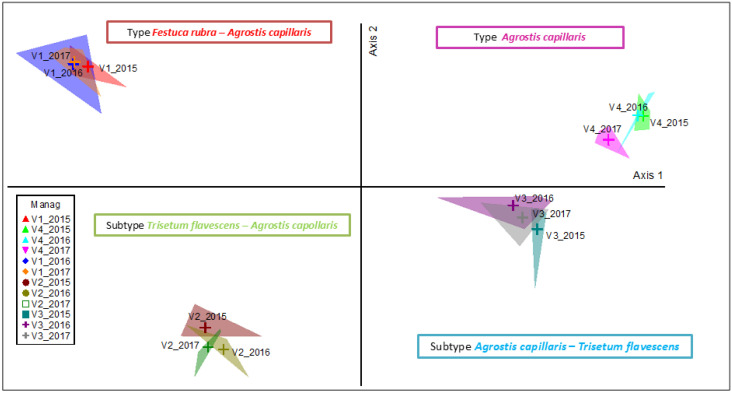
PCoA of grassland types modified by the application of mineral inputs. Legend. Manag—management; V1—control; V2—N_50_P_25_K_25_; V3—N_100_P_50_K_50_; V4—N_150_P_75_K_75_; 2015, 2016, 2017 = the three experimental years; R1, R2, R3, R4 = the four replications; management codes: 1, 2, 3—V1; 4, 5, 6—V2; 7, 8, 9—V3; 10, 11, 12—V4; first management correspond to year 2015, second management correspond to year 2016 and third management correspond to year 2017.

The application of mineral fertilization caused major changes in the floristic composition of the grassland (Figure 2). The two main axes of ordination together explain 90.2% of the total variation in the floristic composition (Table 1). Axis 1 explains most of the variation (89.6%) and shows a clear separation between recorded communities as shaped by the fertilization level. On the left side is the control variant (V1), characterized by the *Festuca rubra—Agrostis capillaris* grassland type, with a stable composition in the three years analyzed. In the lower left quadrant, the low-input variant (V2—N_50_P_25_K_25_) is highlighted, and the community with the *Trisetum flavescens—Agrostis capillaris* subtype reflect a moderate change in the community structure under this reduced fertilization. On the right side of the ordination, the variants with medium- and high-input fertilization are grouped: V3 (N_100_P_50_K_50_), characterized by the subtype *Agrostis capillaris—Trisetum flavescens* and V4 (N_150_P_75_K_75_), where the type *Agrostis capillaris* dominates.

Axis 2, which explains only 0.6% of the total variation, contributes in a small proportion to the differentiation of the communities, which confirms that the trophic gradient induced by mineral fertilization (captured on axis 1) is the main factor of changes in floristic composition.

The distance between the control variants from 2015, 2016 and 2017 was determined with MRPP in order to assess the influence of climate and treatments on the recorded coverage. T is the MRPP test statistic which calculates the separation among the groups. The more negative the T values are, the stronger the separation is. The T values were T = −0.063, *p* < 0.05 for V12015 vs. V12016, and T = −0.342, *p* < 0.05 for V12015 vs. V12017 (Table 2), which indicates no significant floristic distance. A is the MRPP agreement statistic which calculates the homogeneity within groups. The A values fall between 0 (indicating heterogeneity) and 1 (indicating homogeneity). The A value was A = −0.001 for V12015 vs. V1 2016, which is to be expected since agreement values are commonly below 0.1 in community ecology [16]. The MRPP analysis shows that the variability between years lacks significant changes, which justifies the use of cumulative data in the following analyses. This also highlights that the observed differences are due to the fertilization effect, which is the dominant and statistically significant driver, outweighing any interannual climatic effects within the studied period. Climate has only marginal influences on the vegetal coverage and on the grassland type.

**Figure 3:**
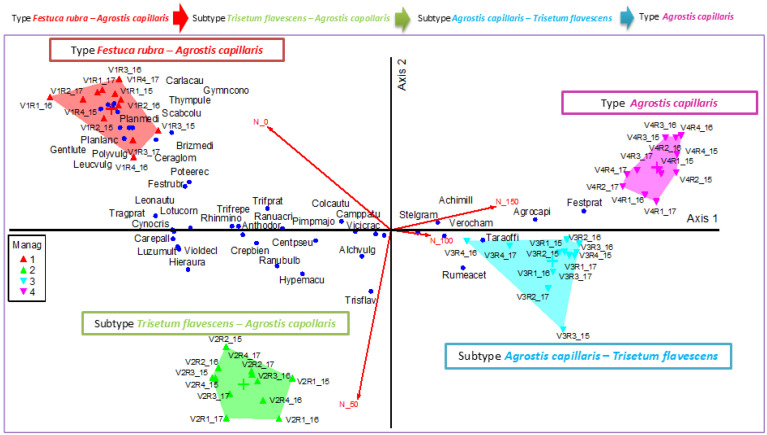
V1—control (N_0); V2—N_50_P_25_K_25_ (N_50); V3—N_100_P_50_K_50_ (N_100); V4—N_150_P_75_K_75_ (N_150); 15, 16, 17 = the three experimental years; R1, R2, R3, R4 = the four replications.

For the interpretation of the results in Figure 3, we used the data accumulated over the three years of observations, which is equivalent to 12 replications for each experimental variant. This approach provides a higher degree of statistical robustness and confidence in the exploration of the identified differences. The PCoA analysis based on the fertilization vectors and their correlation with the ordination axes (Table 3) shows that axis 1 is closely correlated with high input levels (V3 and V4, significant negative correlations), which explains the positioning of these treatments on the right side of the graph. Both treatments are associated with nitrophilous species such as *Agrostis capillaris*, *Rumex acetosa* and *Taraxacum officinale*. In contrast, the control variant (V1) is negatively correlated with Axis 1, but the recorded communities are characterized as oligotrophic and dominated by *Festuca rubra*, *Carlina acaulis* and *Carex pallescens*, typical for stable HNV grasslands.

Axis 2 is positively associated with the low-input variant (V2, *p* < 0.001), which indicates the intermediate positioning of this treatment between the control and high inputs. The specific V2 community is characterized by the appearance of mesotrophic species, such as *Trisetum flavescens* and *Hypericum maculatum* (which marks a moderate transition in the community structure.

Therefore, the treatment vectors highlight a clear trophic gradient: from oligotrophic and diverse communities (V1) to intermediate communities with partially maintained stability (V2) and further to simplified and unstable phytocoenoses dominated by *Agrostis capillaris* (V3 and V4).

Legend:—Carlacau-*Carlina acaulis*, Gymncono-*Gymnadenia conopsea*, Thympule-*Thymus pulegioides*, Scabcolu-*Scabiosa columbaria*, Planmedi-*Plantago media*, Planlanc-*Plantago lanceolata*, Gentlute-*Gentiana lutescens*, Polyvulg-*Polygala vulgaris*, Brizmedi-*Briza media*, Ceraglom-*Cerastium holesteoides*, Leucvulg-*Leucanthemum vulgare*, Poteerec-*Potentilla erecta*, Festrubr-*Festuca rubra*, Leonautu-*Leontodon autumnalis*, Tragprat-*Tragopogon pratensis*, Cynocris-*Cynosurus cristatus*, Lotucorn-*Lotus corniculatus*, Rhinmino-*Rhinanthus minor*, Trifrepe-*Trifolium repens*, Trifprat-*Trifolium pratense*, Ranuacri-*Ranunculus acris*, Anthodor-*Anthoxanthum odoratum*, Pimpmajo-*Pimpinella major*, Colcautu-*Colchicum autumnale*, Camppatu-*Campanula patula*, Vicicrac-*Vicia cracca*, Carepall-*Carex pallescens*, Luzumult-*Luzula multiflora*, Hieraura-*Hieracium aurantiacum*, Violdecl-*Viola declinata*, Crepbien-*Crepis biennis*, Ranubulb-*Ranunculus bulbosus*, Hypemacu-*Hypericum maculatum*, Centpseu-*Centaurea pseudophrygia*, Alchvulg-*Alchemilla vulgaris*, Trisflav-*Trisetum flavescens*, Rumeacet-*Rumex acetosella*, Taraoffi-*Taraxacum officinale*, Stelgram-*Stellaria graminea*, Achimill-*Achillea millefolium*, Verocham-*Veronica chamaedrys*, Agrocapi-*Agrostis capillaris* and Festprat-*Festuca pratensis*.

MRPP analysis confirmed the differences highlighted by PCoA, with all comparisons between variants being significant (*p* < 0.001, Table 4). Negative values of the T statistic (−14.393 to −15.836) indicated a real and consistent separation between treatments. The A coefficient ranged from 0.343 to 0.730, with the largest differences being between the control and the high-input variant (V1 vs. V4, A = 0.730) and between the control and medium input (V1 vs. V3, A = 0.645). In contrast, the lowest value (V3 vs. V4, A = 0.343) suggests that the high-input variants were similar to each other, both being dominated by *Agrostis capillaris*, which shows that medium and high inputs lead to convergent phytocoenoses, uniformed by nitrophilous species (Table 5).

### 2.3. Species Reaction to the Gradient of Applied Inputs

The analysis of the correlation between species and the ordination axes shows that the application of mineral fertilizers generates clear differences in the distribution of species in the phytocoenoses (Table 5). Axis 1 separates the communities associated with the control (V1) from those associated with high inputs (V3 and V4). A total of 31 species were negatively correlated with axis 1 and positively correlated with axis 2, which shows that these species are favored by the lack of mineral fertilization and are not advantaged by the high doses of N_150_P_75_K_75_ type (V4). Among them, multiple species stand out: *Anthoxanthum odoratum* (*p* < 0.001), *Briza media* (*p* < 0.05), *Carex pallescens* (*p* < 0.001), *Trifolium pratense* (*p* < 0.01), *Carlina acaulis* (*p* < 0.01) and *Gentianella lutescens* (*p* < 0.01). These species can be considered indicators of the oligotrophic character of the grassland and were particularly associated with the control and low-input variants.

On the other hand, certain species were strongly positively correlated with axis 1, which shows an increase in their participation with the application of mineral fertilization at medium and high doses. Thus, *Agrostis capillaris* (*p* < 0.001) is the most favored by intensive fertilization, followed by species *Rumex acetosa* (*p* < 0.001), *Taraxacum officinale* (*p* < 0.01) and *Veronica chamaedrys* (with a certain tendency), which are adapted to nitrophilic conditions.

Axis 2 differentiated, in particular, the low-input variant (V2—N_50_P_25_K_25_), which was associated with mesotrophic species. Among these were *Trisetum flavescens* (*p* < 0.001), whose participation increased under reduced fertilization, and *Hypericum maculatum* (*p* < 0.05). Other species, *Hieracium aurantiacum*, *Luzula multiflora*, *Ranunculus bulbosus* and *Alchemilla vulgaris*, had negative correlations with axis 2, showing a trend which suggests that they prefer low fertilization doses and maintain themselves better in low-input variants.

### 2.4. The Influence of Mineral Fertilizer on Plant Diversity

The control variant (V1) presents maximum values for the number of species (42.33), with a Shannon index of 3.00, an equity of 0.802 and a Simpson index set to 0.919, confirming the character of a stable and diverse HNV grassland. Reduced fertilization (V2—low-input) causes a moderate decrease in the number of species (34.00) and the Shannon index (2.70), but maintains values close to the control for equity (0.765) and the Simpson index (0.896). This comparison shows that reduced inputs do not significantly destabilize the community.

In contrast, medium fertilization (V3—medium-input) accentuates the loss of diversity, with the number of species reducing to 22.00 and the Shannon index to 1.81. The equity decreases to 0.586 and the Simpson index to 0.680, which indicates the simplification of the vegetation cover and the establishment of species with higher tolerance to nutrient input. High fertilization (V4—high-input) generates the lowest values for all analyzed indices: 18.00 species (such as plant species *Agrostis capillaris*, *Rumex acetosa*, *Festuca pratensis*, etc.), H′ = 1.31, E = 0.453 and D = 0.496, confirming the dominance of nitrophilic species and the loss of ecosystem stability. The dominant nitrophilic species is *Agrostis capillaris*. This species is consumed by animals, has medium fooder value, is medium-tolerant to mowing and is medium-tolerant to grazing (Table 6).

The high F test values and statistical significance (*p* < 0.001 for all indices) demonstrate that the differences between the variants are significant and that long-term mineral fertilization profoundly influences the structure and diversity of grassland communities.

## 3. Discussion

The results confirm that long-term mineral fertilization profoundly influences the structure and diversity of HNV grasslands, generating a well-defined trophic gradient. Cluster analysis highlights the transition from diverse communities dominated by *Festuca rubra—Agrostis capillaris* [61] to simplified communities dominated by *Agrostis capillaris*. *Festuca rubra—Agrostis capillaris* grasslands are present over large areas within the Apuseni Natural Park [62]. In general, this type of grassland resists reduced fertilization, maintaining their stability [63]. In the low-input variant (V2—N_50_P_25_K_25_), the grassland shows a dominant community between *Trisetum flavescens—Agrostis capillaris*, suggesting a moderate ecological transition of the community structure towards mesotrophic species with intermediate nutritional requirements [64]. The study by [65] shows that the cumulative effects of fertilization manifest over several decades. In the case of reduced or moderate inputs, plant communities maintain their basic structure in the long term, without rapid losses of dominant and nutrient-tolerant species [66]. In the absence of fertilization, the phytocoenose could return to its initial structure, a phenomenon also reported in other European experiments [67,68,69]. However, over time, even under low inputs, an evolutionary trend towards the *Trisetum flavescens—Agrostis capillaris* subtype is observed [70,71,72]. In contrast, medium and high fertilization (V3–V4) led to the dominance of *Agrostis capillaris*, a species known for its plasticity and nitrogen tolerance [73,74,75]. Communities of this type show an obvious simplification in flora and an increase in the proportion of nitrophilic species, confirming the observations of [76,77,78]. Therefore, cluster analysis reveals not only a statistical differentiation between treatments, but also a predictable ecological trend: the diverse and stable *Festuca rubra*-type communities are gradually replaced by simplified communities dominated by *Agrostis capillaris*, which raises questions regarding the sustainability of the continuous application of mineral fertilization in mountain HNV grasslands.

Both PCoA and MRPP analyses confirmed the differences between treatments, showing a clear separation of the experimental variants based on the fertilization level, but without significant variations between years. To obtain this general view, it is justified to use cumulative data (12 replications/variant) to provide statistical robustness to the results. The identified gradient is consistent with similar studies conducted in Apuseni [43,79] and with European meta-analyses on nitrogen fertilization [80].

Correlations between species and PCoA axes highlighted a set of indicator species for each fertilization level. The control communities (V1) were characterized by oligotrophic species (*Festuca rubra*, *Anthoxanthum odoratum*, *Briza media*, *Carex pallescens*, *Carlina acaulis*), plant species that are representative for HNV grasslands [17,81]. Reduced fertilization (V2) favored the emergence of mesotrophic species (*Trisetum flavescens*, *Hypericum maculatum*), indicating a moderate transition [4,82]. Although the correlations were not statistically significant for *Hieracium aurantiacum*, *Luzula multiflora*, *Ranunculus bulbosus* and *Alchemilla vulgaris*, the literature shows that these species are characteristic for oligotrophic or poorly fertilized mountain grasslands, being frequently mentioned as indicator species in traditionally mowed or low-fertilization systems [83,84]. This confirms the trend observed in our analysis, where these species negatively correlated with axis 2, suggesting an affinity for low inputs. Medium and high inputs led to the dominance of *Agrostis capillaris* and other nitrophilous species (*Rumex acetosa*, *Taraxacum officinale*), in line with the observations of other authors [53,73,85].

The diversity indices—Shannon, Evenness and Simpson—supported the trends observed in plant communities. The control presented the highest values, confirming the diverse and stable character of the oligotrophic communities [14]. Low fertilization (V2—low-input) caused a moderate decrease in diversity, but without drastic simplifications of the community structure, suggesting that this level of management could be compatible with the maintenance of biodiversity in HNV grasslands, as also highlighted by [68]. High inputs significantly reduced diversity and evenness, indicating a loss of ecosystem stability [41,86].

The results highlight a clear succession of plant community types as nutrient input increases. For V1 (control, without fertilization) the results show the presence of diverse communities, dominated by *Festuca rubra* and oligotrophic species (*Carex pallescens*, *Carlina acaulis*, *Anthoxanthum odoratum*), and stability indices [87,88]. These grasslands represent a classic form of HNV associated with extensive management. The V2 (low-input, N_50_P_25_K_25_) sustain moderately modified phytocoenoses, with the appearance and increase in the share of *Trisetum flavescens* and other mesotrophic species. Diversity is slightly reduced, but the equity and stability of the community remain high, suggesting an acceptable compromise between agronomic quality, pastoral value and biodiversity conservation [89]. The increase in the fertilization scenario which is associated with V3 (medium-input, N_100_P_50_K_50_) leads to an obvious transition toward simplified communities. These communities are dominated by *Agrostis capillaris* and accompanied by nitrophilous species such as *Rumex acetosa* or *Taraxacum officinale*. Diversity and equity indices decrease significantly, confirming the loss of ecosystem stability. The maximum dose of fertilizer form V4 (high-input, N_150_P_75_K_75_) reorganizes the phytocoenoses to a highly simplified one, dominated almost exclusively by *Agrostis capillaris*. The low values of all indices show the loss of biodiversity and the establishment of an unstable community, with reduced ecological functionality.

The long-term cumulative effects are an important research topic for grassland stability and diversity, with long-term trial studies showing that low inputs maintain the basic structure of communities for decades [50,65], while medium and high inputs accelerate the transition to simplified and unstable communities. Overall, the research highlights two main types of communities: (i) diverse and resilient communities dominated by *Festuca rubra* (control and low-input scenarios) and (ii) simplified communities dominated by *Agrostis capillaris* (medium- and high-input scenarios).

Our results highlight the importance of maintaining extensive management or low fertilization, compatible with biodiversity conservation objectives and the requirements of the Common Agricultural Policy on High-Nature-Value grasslands. At the same time, this study demonstrates the relevance of long-term research for understanding ecological processes and for substantiating strategies for the sustainable use of mountain resources. Although our study highlights the response of phytocoenoses to different levels of fertilization, it should be emphasized that in HNV grasslands included in the Natura 2000 network, the use of mineral fertilizers is prohibited, which limits the direct applicability of the results and emphasizes the need to identify acceptable alternatives. Overall, the results show that HNV mountain grasslands can be maintained as stable only through extensive management or under reduced inputs, while agricultural intensification inevitably leads to biodiversity loss and simplification of plant communities. These conclusions, congruent with European trends, emphasize the relevance of long-term experiments for the substantiation of agricultural policies compatible with conservation objectives.

The results of the experiment show that reduced inputs can be compatible with maintaining the biodiversity and stability of mountain grasslands, offering a compromise between productivity and conservation [90,91]. In HNV grasslands included in the Natura 2000 network, the use of mineral fertilizers is prohibited; therefore, the recommendations should be interpreted as scientific benchmarks and translated into practice by identifying acceptable alternative inputs (composted manure, natural organic amendments, mulching). Sustainable management should privilege reduced fertilization or its substitution with organic sources to maintain biodiversity and reduce the ecological risks associated with intensification [92,93]. Agricultural policies and measures under the CAP should integrate these results to support biodiversity conservation and long-term stability of HNV grasslands [94,95,96,97]. In addition to the prohibition of the use of mineral fertilizers in HNV Natura 2000 grasslands, our results suggest that alternative inputs (composted manure, organic amendments or mulching), can be integrated into accepted agroecological practices, maintaining the biodiversity and stability of phytocenoses and representing viable solutions that can capitalize on the scientific results obtained in this study [98,99].

## 4. Materials and Methods

### 4.1. Study Area

The experiment was conducted in Gârda de Sus, Alba County, Apuseni Mountains (Western Carpathians, Romania; 46°29′26.4″ N 22°48′53.7″ E), on a semi-natural permanent grassland located at an average altitude of 1130 m with a slope of approximately 5%. The soil had the typology of red, weakly skeletal preluvosol, with predominantly southeast exposure, characterized by moderate fertility and low content of humus and nutrients, which determined its limited natural productivity [100]. The multiannual average temperature was 5.1 °C, and the annual precipitation amounted to approx. 1042 mm. Management consisted of annual mowing, carried out at the beginning of July, and the resulting grass was removed from the experimental plots. The analyzed data covered three experimental years (2015–2017) but reflected the cumulative effect of mineral fertilization applied annually since 2001, the year of the initiation of the experiment. Before the initiation of the experiment, in 2001, the meadow was used in a mixed exploitation system. It was mowed once a year, and in the autumn, it was grazed extensively with cattle, with a stocking rate of approximately 0.2–0.4 UVM/ha [49,101,102]. Regarding the management applied prior to the installation of the experiment, the meadow surface was not fertilized, with the nutrient supply coming exclusively from the manure of the animals grazing in an extensive system. Starting with 2001, the meadow maintenance was performed only by mowing, with the removal of the resulting biomass [49].

Regarding the temperature situation recorded at the Ghețari station, in the last 17 years, the following aspects can be observed: the multiannual average was around 5.80 °C, with a maximum value of 7.7 °C recorded in 2012 [49,103] and 2015 (Table 7), respectively, and the minimum value of 3.2 °C recorded in 2005 [49,104]. Table 7 also shows the increasing trend of the average annual temperature, especially in the period 2015–2017 (experimental years). Therefore, the context of climatological changes in the study area brings important effects/changes on the floristic diversity of the oligotrophic meadows in the Apuseni Mountains [43,49].

Regarding the average annual precipitation values recorded at the Ghețari weather station, it was found that the multiannual average (over 17 years) had the value of 1042.1 mm, with the maximum being recorded in 2001 (1553 mm) and the minimum recorded in 2012 (687 mm), which was considered the driest year in our study area. Comparing the 3 experimental years (2015–2017 period) with the multiannual average, it can easily be seen that the decreasing trend in precipitation values is also complemented by the increasing temperature values (Table 8); therefore a continuously changing picture is foreseen from a climatological point of view [43,49,105].

The study area is part of the Natura 2000 network, being included in the ROSCI0002 Apuseni site, which underlines the importance of protecting rare habitats and species in the area. More precisely, the study area is part of the mountain meadow habitat 6520, Natura 2000 Site, in the Apuseni Natural Park, contributing to the understanding of the complexity of the ecosystem and the implementation of effective conservation measures for this habitat [22,81,106]. The mountain meadow habitat 6520 in the Apuseni Natural Park is particularly important due to its floristic diversity and high ecological value. In this area, the traditional use of meadows has been preserved, which has allowed the maintenance of a habitat with an extraordinary flora, where rare species are found, such as *Lilium jankae*, *Allium victoriale*, *Centaurea kotschyana*, *Trollius europaeus*, *Anemone narcissifolia*, *Centaurea mollis*, *Delphinium elatum*, *Dianthus barbatus ssp. Compactus* and *Hypericum richerii* ssp. *grisebachii* [22,105,107,108].

### 4.2. Experimental Design

A long-term experiment, established in 2001, was used to collect the data and to analyze the vegetation trends. A completely randomized block design was used, with 4 treatment variants and 4 replications, on a total of 16 adjacent plots, each with an area of 10 m^2^ (2 × 5 m). The variants were as follows (kg/ha): V1—control variant (unfertilized), V2—N_50_P_25_K_25_ (N 50 kg ha^−1^, P_2_O_5_ 25 kg ha^−1^, K_2_O 25 kg ha^−1^), V3—N_100_P_50_K_50_ (N 100 kg ha^−1^, P_2_O_5_ 50 kg ha^−1^, K_2_O 50 kg ha^−1^) and V4—N_150_P_75_K_75_ (N 150 kg ha^−1^, P_2_O_5_ 75 kg ha^−1^, K_2_O 75 kg ha^−1^). Mineral fertilizers were administered annually, in early spring, before the start of active vegetation. We used granular NPK 15:15:15 and granular ammonium nitrate for N completion. They were applied once a year in each experimental variant. Mowing was carried out at the optimal time (when the grasses were in the phenological flowering phase), and the vegetal material was removed outside the experiment. The entire experimental field was fenced to prevent access by wild or domestic animals.

Regarding the floristic composition of the meadow before the installation of the experiment, a detailed study of the meadow vegetation was carried out in 2001. Within this, all plant species present in the experimental area were identified, as well as their degree of coverage, thus establishing the initial floristic structure (Moment 0), before the delimitation of the plots and the application of the experimental treatments (installed later in 2001). The complete list of identified species, together with the corresponding values of coverage, is available in the Supplementary Materials (Supplementary Table S2).

### 4.3. Floristic Composition

**Figure 4:**
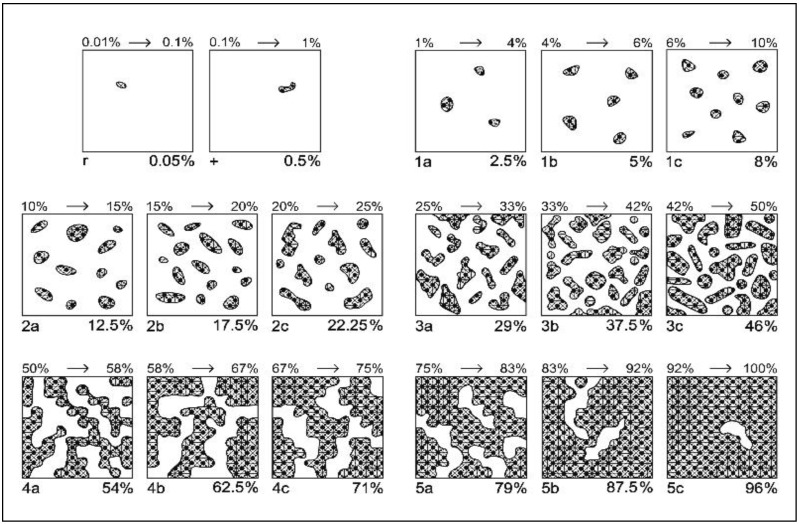
Modified Braun-Blanquét scale for grasslands based on species coverage (after [86]). Legend: 1 to 5 indicate the class of coverage; a, b, c indicate the sub-note of each class.

The floristic composition was assessed using the Braun-Blanquét method, modified for mountain grasslands by [86] (Figure 4, Table 9). A similar approach was also used in recent studies in the Eastern Alps (e.g., case studies in three mountain regions), where the Braun-Blanquet scale was subdivided into finer classes to increase the resolution of the assessments and the accuracy of estimating species cover in alpine grasslands [109]. This modification is justified by the need for a more precise discrimination of cover variations, especially in communities with a complex structure and high diversity, such as HNV mountain grasslands. The cover estimation was carried out in early July, when most grasses were in the flowering phase. This harvesting period ensured relevance for comparisons regarding vegetation structure [110,111]. Floristic studies were carried out in each experimental variant on the entire 10 m^2^ area, with 4 repetitions each, and this paper presents floristic data over a period of 3 years, which reflects the cumulative effect of mineral fertilization after 17 years of application. Specialized determiners and instruments adapted to them were used to determine plant species. For scientific nomenclature, resources and international databases recognized in botany, such as POWO (Plants of the World Online) and Euro+Med PlantBase, were used. To ensure the accuracy of the data, all experimental variants were fenced, so that other animals could not enter and influence the results of the study. The optimal time for determining the floristic composition in the study area is July, when the grasses are in bloom, which ensures the correctness of the composition of the floristic study, as shown by other studies conducted on semi-natural meadows [110,111].

### 4.4. Data Analysis

Multivariate analyses were performed with PC-ORD version 7 [112,113] a software widely used in community ecology for ordination, classification and testing differences between plant groups. This software has been applied in numerous recent studies, including for the analysis of plant and animal community structures, demonstrating its robustness and flexibility in handling complex ecological data [114,115].

Cluster analysis was used to group the recorded communities based on their similarity or differences (dissimilarity) to define grassland types [116,117]. The cluster analysis used the Sørensen similarity index (Bray–Curtis) and the UPGMA (Group Average linkage) clustering method, considered a good standard in plant community analysis and applied in numerous recent studies to delimit ecological and phytosociological groups [118,119,120]. The dendrogram cutoff level was set at approximately 90% remaining information, which allowed the obtaining of clusters with ecological and phytosociological relevance.

Ordination analysis was necessary to highlight the trophic gradient and the separation of grassland types according to treatment and was performed with PCoA (Principal Coordinates Analysis) using the Bray–Curtis distance. This method is frequently applied in ecology to compare plant or microbial communities based on composition. The choice of PCoA is justified by the fact that it provides a unique and reproducible solution, allowing direct interpretation of differences or similarities between samples, unlike iterative methods such as NMDS, which can produce variable solutions. Recent studies confirm the applicability of this approach: for example, ref. [121] used PCoA + Bray–Curtis to differentiate soil fauna communities between grazed and ungrazed grasslands, and [122] highlighted changes in the structure of microbial communities induced by fertilization using the same type of analysis. Ref. [123] showed that the type of fertilization and the planting method influence the structure of fungal and bacterial communities, visualized by PCoA based on the Bray–Curtis distance.

For each experimental variant, vectors corresponding to the fertilization levels were drawn (N0-V1-control variant-zero-input; N_50_-V2-N_50_P_25_K_25_-low-input (applied in each experimental year); N_100_-V3-N_100_P_50_K_50_-*medium-input* (applied in each experimental year); N_150_-V4-N_150_P_75_K_75_-high-input (applied in each experimental year). This classification reflects the reporting to the thresholds established by the Nitrates Directive (170 kg N/ha/year), as well as the ecological response of the vegetation observed in the field. The procedure for classifying the experimental treatments by input categories (“Zero-input”, “Low-input”, “Medium-input”, “High-input”) follows common practices in the experimental ecology literature, where treatments with different fertilization levels (or nutrient inputs) are compared for effects on plant diversity and productivity [122,123,124]. Vectors were normalized and their significance was tested by permutations (*n* = 999). PC-ORD offers options for ordination based on Bray–Curtis distances, overlays or joint plots and randomization tests, which makes the choice to use vectors corresponding to fertilization levels consistent with good methodological practices [113,125]. The first two ordination axes were retained for interpretation, as they together explained over 90% of the variation in floristic composition.

To test the differences between the groups identified by cluster analysis and by ordination, the MRPP (Multi-Response Permutation Procedure) was applied. This non-parametric method compares the intragroup similarity with that between groups, providing three main indicators: the A coefficient, which expresses the degree of homogeneity within groups (positive values indicate greater consistency than would be expected by chance); the T test, which describes the degree of separation between groups (the more negative the T value, the stronger the separation); and the *p* value, which evaluates the statistical significance of the differences. The significance threshold was set at α = 0.05, which is the conventional standard in community ecology. The use of MRPP is frequently reported in the recent literature, including in studies validating the separation of plant and animal communities according to environmental factors or experimental treatments [114,115].

### 4.5. Diversity Index Analysis

For each experimental treatment, α-diversity indices were calculated: specific richness (S), Shannon–Wiener index (H′), species evenness (E) and Simpson index (D). These indices are frequently used in ecological studies of grasslands and are considered robust tools for assessing community diversity and balance [126,127,128,129]. Ref. [130] applied these indices to characterize vegetation diversity based on remote sensing images, and [128] demonstrated significant differences between communities in protected and unprotected areas, confirming the relevance of these methods in ecosystem analysis. The calculation formulas are standardized in community ecology [91] and were applied based on the relative abundance of species. To compare the differences between treatments, one-way ANOVA was used, and the separation of means was performed by the LSD test at a significance threshold of *p* < 0.05.

## 5. Conclusions

Long-term mineral fertilization profoundly influences the structure and diversity of HNV grasslands, determining a clear trophic gradient: from diverse and stable communities dominated by *Festuca rubra* to simplified and unstable communities dominated by *Agrostis capillaris*. The low-input variant (N_50_P_25_K_25_) maintained the biodiversity and stability of the communities, confirming the resilience of the *Festuca rubra—Agrostis capillaris* grassland type to low inputs. In contrast, medium and high inputs led to the loss of oligotrophic species and the dominance of nitrophilic species. Multivariate analyses confirmed the robustness of this trophic gradient, demonstrating that the differences were determined by the level of fertilization and not by interannual climatic variations. The results emphasize the relevance of long-term experiments for understanding ecological processes and for substantiating sustainable management strategies for mountain HNV grasslands. The paper highlights the unique importance of long-term experiments for the mountain regions of Romania, providing a solid scientific basis for guiding agricultural policies and for maintaining HNV grasslands. These findings support the integration of long-term experiments into the strategies of the Common Agricultural Policy to guide the sustainable management of HNV grasslands in the Carpathians.

## Figures and Tables

**Table 1:** Importance of axis.

Axis	Degree of Participation (r)	Cumulative
1	0.896	0.896
2	0.066	0.962

Note: r—correlation coefficient between ordination distances and original distances in the n-dimensional space.

**Table 2:** Comparison of the floristic composition changes due to mineral inputs (MRPP).

Treatments	T	A	*p* Value
V1_2015 vs. V1_2016	−0.063	0.001	0.429
V1_2015 vs. V1_2017	−0.342	0.010	0.330
V2_2015 vs. V2_2016	−0.555	0.014	0.262
V2_2015 vs. V2_2017	−0.592	0.016	0.263
V3_2015 vs. V3_2016	−0.204	0.009	0.326
V3_2015 vs. V3_2017	0.950	−0.038	0.829
V4_2015 vs. V4_2016	0.825	−0.058	0.808
V4_2015 vs. V4_2017	−1.216	0.063	0.115

Note: T—statistic test, A—statistic agreement, V1—control; V2—N_50_P_25_K_25_; V3—N_100_P_50_K_50_; V4—N_150_P_75_K_75_.

**Table 3:** Correlation of fertilization treatments with PCoA axes.

Experimental Factors	Axis 1		Axis 2	
	*r*	Significance	*r*	Significance
V1 Control	0.660		−0.601	
V2 50_N_25_P_25_K_	0.344	*	0.771	***
V3 100_N_50_P_50_K_	−0.372	*	0.140	ns
V4 150_N_75_P_75_K_	−0.608	***	−0.289	ns

Note: r—correlation coefficient between ordination distances and original distances in the n-dimensional space. Significance: *p* < 0.001—***; *p* < 0.5—*; ns—not significant.

**Table 4:** Comparison of the floristic composition changes due to mineral inputs (MRPP).

Treatments	T	A	*p*
V1 vs. V2	−15.333	0.398	<0.001
V1 vs. V3	−15.714	0.645	<0.001
V1 vs. V4	−15.836	0.730	<0.001
V2 vs. V3	−15.588	0.603	<0.001
V2 vs. V4	−15.808	0.718	<0.001
V3 vs. V4	−14.393	0.343	<0.001

Note: V1—control; V2—N_50_P_25_K_25_; V3—N_100_P_50_K_50_; V4—N_150_P_75_K_75_. Note: T—statistic test, A—statistic agreement.

**Table 5:** Correlation of species with the ordination axis.

Species	Axis 1				Axis 2			
	r	r-sq	tau	Signif.	r	r-sq	tau	Signif.
*Agrostis capillaris* L.	0.983	0.966	0.901	***	0.160	0.025	0.076	ns
*Anthoxanthum odoratum* L. s. str.	−0.792	0.627	−0.694	***	0.049	0.002	0.010	ns
*Briza media* L.	−0.462	0.213	−0.545	**	0.429	0.184	0.527	*
*Cynosurus cristatus* L.	−0.760	0.577	−0.574	***	0.005	0.000	0.082	ns
*Festuca pratensis* Huds. s. l.	0.230	0.053	0.134	ns	0.049	0.002	0.052	ns
*Festuca rubra* L.	−0.886	0.786	−0.764	***	0.397	0.157	0.145	*
*Trisetum flavescens* (L.) P. Beauv.	−0.150	0.023	−0.124	ns	−0.953	0.908	−0.811	***
*Carex pallescens* L.	−0.937	0.877	−0.711	***	−0.077	0.006	0.041	ns
*Luzula multiflora* (Ehrh.) Lej.	−0.957	0.915	−0.715	***	−0.151	0.023	−0.017	ns
*Lotus corniculatus* L.	−0.613	0.375	−0.662	***	0.015	0.000	−0.010	ns
*Trifolium pratense* L.	−0.535	0.287	−0.445	**	0199	0.040	0.265	ns
*Trifolium repens* L.	−0.748	0.560	−0.582	***	0.039	0.001	0.154	ns
*Vicia cracca* L. s. str.	−0.036	0.001	−0.059	ns	−0.052	0.003	−0.032	ns
*Achillea millefolium* L.	0.200	0.040	0.053	ns	0.071	0.005	0.109	ns
*Alchemilla vulgaris* L.	−0.303	0.092	−0.209	ns	−0.558	0.311	−0.419	ns
*Campanula patula* L.	−0.088	0.008	−0.246	ns	−0.043	0.002	−0.014	ns
*Carlina acaulis* L.	−0.537	0.288	−0.476	**	0.472	0.223	0.427	**
*Centaurea pseudophrygia* C. A. Mey.	−0.659	0.434	−0.529	***	−0.189	0.036	−0.087	ns
*Cerastium holosteoides* Fr.	−0.439	0.193	−0.379	*	0.411	0.169	0.360	*
*Colchium autumnale* L.	−0.335	0.112	−0.349	*	0.130	0.017	0.089	ns
*Crepis biennis* L.	−0.401	0.160	−0.446	*	−0.078	0.006	0.138	ns
*Gentianella lutescens* (Velen.) Holub	−0.513	0.264	−0.441	**	0.492	0.242	0.448	**
*Gymnadenia conopsea* (L.) R. Br. s. l.	−0.650	0.423	−0.571	***	0.606	0.367	0.559	***
*Hieracium aurantiacum* L.	−0.467	0.218	−0.345	**	−0.189	0.036	−0.104	ns
*Hypericum maculatum* Crantz s. str.	−0.472	0.223	−0.329	**	−0.486	0.237	−0.236	**
*Leontodon autumnalis* L.	−0.712	0.507	−0.579	***	0.090	0.008	0.151	ns
*Leucanthemum vulgare* Lam. s. str.	−0.698	0.487	−0.575	***	0.564	0.318	0.335	**
*Pimpinella major* (L.) Huds.	−0.416	0.173	−0.383	*	−0.025	0.001	−0.042	ns
*Plantago lanceolata* L.	−0.640	0.409	−0.621	***	0.532	0.283	0.340	**
*Plantago media* L.	−0.665	0.442	−0.631	***	0.547	0.299	0.340	**
*Polygala vulgaris* L. s. l.	−0.528	0.278	−0.588	**	0.381	0.145	0.440	*
*Potentilla erecta* (L.) Raeusch.	−0.622	0.387	−0.703	***	0.314	0.098	0.024	ns
*Ranunculus acris* L.	−0.604	0.365	−0.557	***	0.020	0.000	0.057	ns
*Ranunculus bulbosus* L.	−0.449	0.201	−0.494	*	−0.295	0.087	−0.197	ns
*Rhinanthus minor* L.	−0.404	0.163	−0.474	*	−0.026	0.001	−0.146	ns
*Rumex acetosa* L.	0.461	0.212	0.366	**	−0.504	0.254	−0.497	**
*Scabiosa columbaria* L.	−0.501	0.251	−0.611	**	0.398	0.158	0.414	*
*Stellaria graminea* L.	0.183	0.034	0.155	ns	−0.032	0.001	−0.022	ns
*Taraxacum officinale* Weber s. l.	0.598	0.358	0.524	**	−0.132	0.017	−0.185	ns
*Thymus pulegioides* L. s. l.	−0.518	0.268	−0.578	**	0.471	0.221	0.564	**
*Tragopogon pratensis* L. s. l.	−0.754	0.568	−0.556	***	−0.008	0.000	0.077	ns
*Veronica chamaedrys* L. s. str.	0.294	0.087	0.192	ns	−0.063	0.004	−0.124	ns
*Viola declinata* L.	−0.874	0.764	−0.645	***	−0.160	0.026	−0.040	ns

Note: r—correlation coefficient; r-sq—determination coefficient. Significance: *p* < 0.001—***; *p* < 0.01—**; *p* < 0.5—*; ns—not significant.

**Table 6:** The influence of mineral fertilizer on plant diversity.

Variant	Species no. (S)	Shannon (H′)	Evenness (E)	Simpson (D)
V1 (Control)	42.33 ± 0.49 a	3.00 ± 0.08 a	0.802 ± 0.025 a	0.919 ± 0.016 a
V2 (Low-input)	34.00 ± 0.00 b	2.70 ± 0.05 b	0.765 ± 0.015 a	0.896 ± 0.008 a
V3 (Medium-input)	22.00 ± 0.00 c	1.81 ± 0.14 c	0.586 ± 0.046 b	0.680 ± 0.048 b
V4 (High-input)	18.00 ± 0.00 d	1.31 ± 0.20 d	0.453 ± 0.070 c	0.496 ± 0.078 c
F test	24,928.00	416.53	132.76	445.97
*p*.val	*p* < 0.001	*p* < 0.001	*p* < 0.001	*p* < 0.001

**Table 7:** The monthly average temperatures recorded at Gheţari weather station (2015–2017).

Year	Months												Average
	I	II	III	IV	V	VI	VII	VIII	IX	X	XI	XII	
2015	−0.8	2	4.7	3.8	10.8	13.5	16.5	16.5	12.7	7.7	3.4	−0.2	7.7
2016	−5.4	0.1	1	8	8.7	14.8	16	15.3	10.9	4.6	0.4	−5.4	5.7
2017	−1.8	6	8.3	6.4	10	14.6	15.5	15.3	10.7	7.9	0.6	−3.2	7.5
**Average values for the period 2001–** **2017**													
2001–2017	−4.5	−2.7	0.2	5.3	10.5	14.5	15.9	15.4	11.3	6.0	1.4	−3.1	5.8

**Table 8:** The monthly average precipitations recorded at Gheţari weather station (2015–2017).

Year	Months												Total
	I	II	III	IV	V	VI	VII	VIII	IX	X	XI	XII	
2015	32.6	14.2	23.6	48.6	69.4	78.2	33.8	95	124	38.8	98.4	49.8	706.4
2016	120	111.4	79.4	108.4	67	165.4	58.8	49.4	56	86.6	127	0.2	1030
2017	112	0	86	11.4	116.6	95	37	35	88	100	98	36.2	815.2
**Average values for the period 2001–2017**													
2001–2017	67.2	55.9	81.8	77.2	102.4	100.3	137.3	98.4	92.6	86.6	86.5	55.8	1042.1

**Table 9:** Modified Braun-Blanquét scale for assessing the abundance–dominance of plant species, based on classes and sub-classes (after [86]).

Class	Coverage Interval (%)	Class Central Value (%)	Sub-Note	Sub-Interval (%)	Central-Adjusted Value of Sub-Interval (%)
5	75–100	87.5	5c	92–100	96
			5b	83–92	87.5
			5a	75–83	79
4	50–75	62.5	4c	67–75	71
			4b	58–67	62.5
			4a	50–58	54
3	25–50	37.5	3c	42–50	46
			3b	33–42	37.5
			3a	25–33	29
2	10–25	17.5	2c	20–25	22.25
			2b	15–20	17.5
			2a	10–15	12.5
1	1–10	5	1c	6–10	8
			1b	4–6	5
			1a	1–4	2.5
+	0.1–1	0.5	-	-	0.5
r	0.01–0.1	0.05	-	-	0.05

Note: a, b, c indicate the sub-note of each class.

## Data Availability

The data presented in this article are available upon request from the corresponding author and/or primary author.

## Supplementary Materials

The following supporting information can be downloaded at https://www.mdpi.com/article/10.3390/plants14233564/s1, Supplementary Table S1. Floristic composition—average of 2015–2017; Supplementary Table S2. The structure of the type of grassland before the experiment was set up, 2001 (after Pacurar F., PhD thesis 2005).
