# Supplementary material for: Effect of Mineral Fertilization on Vegetation of HNV Pastures in the Apuseni Mountains (Romania)
Source: Plants (Basel). 2025 Nov 21;14(23):3564. doi: 10.3390/plants14233564 (PMC12694496; doi:10.3390/plants14233564)
Supplement: Supplementary file 1 [file plants-14-03564-s001.zip › plants-3972567-supplementary.pdf]

Supplementary files

Supplementary Table S1. Floristic composition – average of 2015-2017

Variants	V1	V2	V3	V4
Species	ADM%	ADM%	ADM%	ADM%
***Agrostis capillaris* L.**	**11.0**	**15.0**	**51.3**	**64.6**
Anthoxanthum odoratum L.	4.4	3.0	1.4	0.5
Briza media L.	0.7	-	-	-
Cynosurus cristatus L.	0.5	0.5	-	-
Festuca pratensis Huds.	-	0.5	-	-
***Festuca rubra* L.**	**19.5**	**7.4**	**1.7**	**0.8**
***Trisetum flavescens* (L.) Beauv.**	**5.5**	**23.0**	**13.3**	**6.8**
Carex pallescens L.	0.5	0.5	-	-
Luzula multiflora (Ehrh.) Lej.	0.5	0.5	-	-
Lotus corniculatus L.	1.2	0.8	0.5	-
Trifolium pratense L.	2.6	1.4	0.9	0.7
Trifolium repens L.	3.3	2.7	0.7	0.5
Vicia cracca L.	1.0	1.0	1.3	0.7
Achillea millefolium L.	0.5	0.5	0.7	0.7
Alchemilla vulgaris L.	4.7	8.0	4.8	4.2
Campanula patula L.	0.5	0.5	0.7	0.5
Carlina acaulis L.	0.5	-	-	-
Centaurea pseudophrygia C. A. Meyer	6.0	6.0	4.1	2.2
Cerastium glomeratum Thuill.	0.5	-	-	-
Colchium autumnale L.	3.0	2.3	2.4	1.4
Crepis biennis L.	0.5	0.8	0.5	0.5
Gentiana lutea L.	0.5	-	-	-
Gymnadenia conopsea (L.) R. Br.	0.5	-	-	-
Hieracium aurantiacum L.	0.5	0.5	-	-
Hypericum maculatum Crantz	2.2	4.0	1.2	1.0
Leontodon autumnalis L.	0.5	0.5	-	-
Leucanthemum vulgare Lam.	2.3	0.5	0.5	0.5
Pimpinella major (L.) Hudson	6.6	5.6	6.0	3.5

Plantago lanceolata L.	2.6	0.5	0.5	-
Plantago media L.	3.8	0.5	0.5	-
Polygala vulgaris L.	0.7	0.5	-	-
Potentilla erecta (L.) Räusch.	2.7	0.8	0.5	-
Ranunculus acris L.	0.5	0.5	0.5	-
Ranunculus bulbosus L.	1.0	1.6	0.8	-
Rhinanthus minor L.	1.2	0.9	0.5	-
Rumex acetosa L.	0.5	2.5	3.0	2.2
Scabiosa columbaria L.	1.4	0.5	-	-
Stellaria graminea L.	0.5	0.5	0.7	0.5
Taraxacum officinale Weber ex Wiggers	0.9	1.9	2.4	3.1
Thymus pulegioides L.	1.2	-	-	-
Tragopogon pratensis L.	0.5	0.5	-	-
Veronica chamaedrys L.	1.4	1.7	3.4	2.2
Viola declinata Waldst. et Kit.	0.5	0.5	-	-

Supplementary Table S2. The structure of the type of grassland before the experiment was set up, 2001 (after Pacurar F., PhD thesis 2005 [49])

**No.**	**Family**	**Species**	**%**
1	**Poacee**	*Agrostis capillaris*	1
2		*Anthoxanthum odoratum*	0.38
3		*Cynosurus cristatus*	0.02
4		*Festuca rubra*	10
5		*Nardus stricta*	2
6	**Fabacee**	*Lotus corniculatus*	0.19
7		*Trifolium pratense*	1
8		*Trifolium repens*	1
9		*Vicia cracca*	0.26
10	**Cyperacee**	*Carex pallescens*	0.07
11	**Juncacee**	*Luzula campestris*	0.03
12	**Apiacee**	*Carum carvi*	0.5
13		*Pimpinella major*	5
14	**Polygalacee**	*Polygala vulgaris*	1
15	**Asteracee**	*Arnica montana*	2
16		*Carlina acaulis*	2
17		*Centaurea pseudophrygia*	5
18		*Gnaphalium sylvaticum*	0
19		*Leontodon hispidus*	0.32

20		*Leucanthemum vulgare*	0.25
21		*Taraxacum officinale*	0.25
22		*Tragopogon pratensis*	0.37
23	**Boraginacee**	*Myosotis nemorosa*	0.08
24	**Brasicacee**	*Cardaminopsis halleri*	0.16
25	**Campanulacee**	*Campanula patula*	0.14
26	**Caryophyllacee**	*Stellaria graminea*	0.06
27		*Cerastium glomeratum*	0.04
28	**Dipsacacee**	*Scabiosa columbaria*	2
29	**Euphorbiacee**	*Euphorbia carniolica*	20
30	**Gentianacee**	*Gentianella lutescens*	0.18
31	**Hyperacee**	*Hypericum maculatum*	10
32	**Lamiacee**	*Prunella vulgaris*	0.39
33	**Liliacee**	*Colchicum autumnale*	1
34	**Linacee**	*Linum catharticum*	0.01
35	**Orchidacee**	*Gymnadenia conopsea*	0.48
36	**Plantaginacee**	*Plantago lanceolata*	2
37		*Plantago media*	2
38	**Polygonacee**	*Rumex acetosa*	0.22
39	**Ranunculacee**	*Ranunculus acris*	2
40		*Ranunculus bulbosus*	2
41	**Rosacee**	*Alchemilla vulgaris*	20
42		*Potentilla erecta*	12
43	**Scrophulariacee**	*Rhinanthus minor*	8
44		*Veronica chamaedrys*	0.16
45	**Violacee**	*Viola canina*	1
46		*Viola declinata*	0.22
